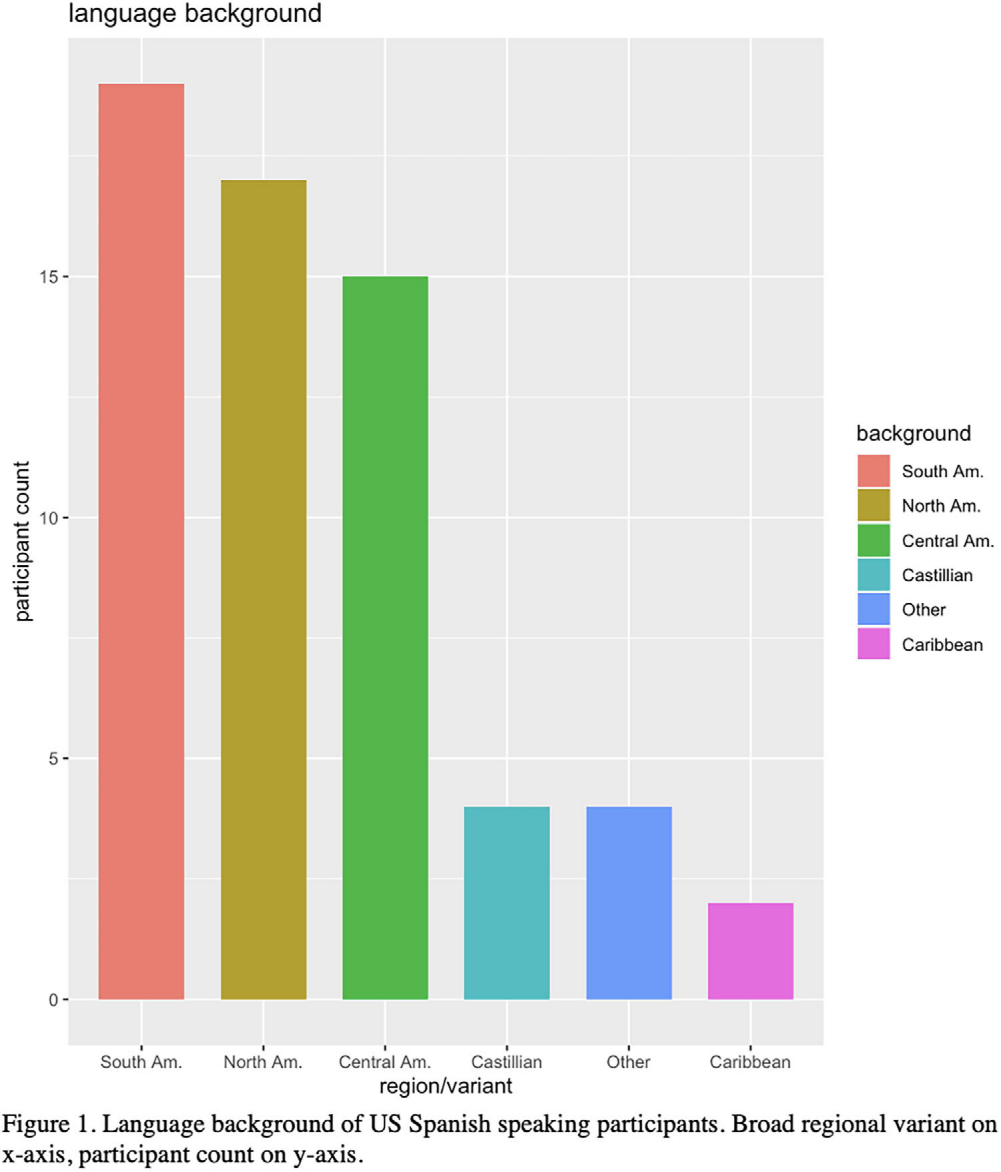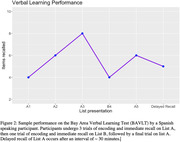# CCAB–Español: The California Cognitive Assessment Battery in Spanish

**DOI:** 10.1002/alz.095572

**Published:** 2025-01-09

**Authors:** Isabella Jaramillo, David L. Woods, Kathleen Hall, Kristin Geraci, Michael Blank, Miranda Miranda, Alejandra Ortiz‐Menchaca, Elloise Garcia, Omar Kahly, Timothy J Herron, Garrett Williams, David K Johnson

**Affiliations:** ^1^ Neurobehavioral Systems, Inc, Berkeley, CA USA; ^2^ Veterans Affairs Northern California Health Care System, Martinez, CA USA; ^3^ University of Chicago, Chicago, IL USA; ^4^ UC Davis Alzheimer’s Disease Center, Walnut Creek, CA USA

## Abstract

**Background:**

By 2060, the incidence of ADRD is predicted to increased 6‐fold in the US Hispanic population (Matthews, Xu et al. 2019). However, cognitive testing of US Hispanics is complicated by limited Spanish‐language test materials and a dearth of examiners fluent in Spanish. Here, we present preliminary results from an automated computerized battery, CCAB–Español, that administers and scores verbal and non‐verbal tests in Spanish.

**Method:**

Spanish‐language equivalents of CCAB‐English test materials were created and presented with text‐to‐speech voices in a Mexican dialect. Participants (n = 61; age 43‐79; 64% women) completed 32 tests over three days, including verbal, visual, memory, and processing speed tests, as well as demographic and psychological questionnaires. Test were administered in participants’ homes and remotely monitored by Spanish‐speaking examiners. Verbal tasks were objectively scored using Consensus Automatic Speech Recognition (CASR). Selected tests were administered twice to assess test‐retest reliability.

**Result:**

Reaction times, response accuracy, error types, and speech metrics were collected and analyzed. Participant satisfaction with the battery was high, with 100% of the participants completing all days of testing. Tests demonstrated excellent test‐retest reliabilities, for example the test‐retest value for delayed recall on a story recall task was .83.

**Conclusion:**

The results from ongoing normative data collection show that CCAB‐Español is an efficient, scalable platform for the comprehensive cognitive assessment of US Spanish speakers.

Matthews, K. A., W. Xu, A. H. Gaglioti, J. B. Holt, J. B. Croft, D. Mack and L. C. McGuire (2019). “Racial and ethnic estimates of Alzheimer’s disease and related dementias in the United States (2015‐2060) in adults aged ≥65 years.” Alzheimer’s & Dementia **15**(1): 17‐24.